# Molecular Mechanisms of Type 2 Diabetes-Related Heart Disease and Therapeutic Insights

**DOI:** 10.3390/ijms26104548

**Published:** 2025-05-09

**Authors:** German Camilo Giraldo-Gonzalez, Alejandro Roman-Gonzalez, Felipe Cañas, Andres Garcia

**Affiliations:** 1 Health Science Doctorate Program, Cardiology, Universidad de Caldas, Manizales 170001, Colombia; 2 Facultad de Medicina, Endocrinology Department, Universidad de Antioquia, Medellin 050010, Colombia; alejoroman@gmail.com; 3 Electrophysiology Unit, Hospital Universitario Fundación Valle del Lili, Cali 760031, Colombia; felipe.canas.orduz@gmail.com; 4 Faculty of Health Sciences, Universidad Tecnológica de Pereira, AA 97, La Julita, Pereira 660003, Colombia; andrmaurgarcia@utp.edu.co

**Keywords:** heart, molecular, diabetes, cardiovascular

## Abstract

Type 2 diabetes is a significant risk factor for cardiovascular disease, particularly coronary heart disease, heart failure, and diabetic cardiomyopathy. Diabetic cardiomyopathy, characterized by heart dysfunction in the absence of coronary artery disease or hypertension, is triggered by various mechanisms, including hyperinsulinemia, insulin resistance, and inflammation. At the cellular level, increased insulin resistance leads to an imbalance in lipid and glucose metabolism, causing oxidative stress, mitochondrial dysfunction, and excess production of reactive oxygen species (ROS). This disrupts normal heart function, leading to fibrosis, hypertrophy, and cardiac remodeling. In diabetic patients, the excessive accumulation of fatty acids, advanced glycation end products (AGEs), and other metabolic disturbances further contribute to endothelial dysfunction and inflammatory responses. This inflammatory environment promotes structural damage, apoptosis, and calcium-handling abnormalities, resulting in heart failure. Additionally, diabetes increases the risk of arrhythmias, such as atrial fibrillation, which worsens cardiac outcomes. New insights into these molecular mechanisms have led to improvements in diabetes management, focusing on mitigating complications and understanding the cellular processes involved. Recent therapeutic advances, such as SGLT-2 inhibitors, have shown promise in addressing the energy imbalance and cardiac dysfunction seen in diabetic cardiomyopathy, offering new hope for better cardiovascular outcomes.

## 1. Introduction

Type 2 diabetes is a highly fatal disease from a cardiovascular perspective [1]. Diabetes-related heart disease has classically been associated only with coronary heart disease, due to its high prevalence; nevertheless, the cellular and inflammatory components play an important role in altering the mechanisms of normal heart function. Heart failure is one of its main manifestations, and damage at the cellular level is triggered by hyperinsulinemia and insulin resistance. Tachycardia-induced cardiomyopathy (TIC) is an underdiagnosed condition that develops ventricular dysfunction and heart failure. It is still necessary to elucidate other mechanisms through which type 2 diabetes affects the cardiovascular system [2].

## 2. Type 2 Diabetes Mellitus and the Cardiomyocyte

The risk of cardiovascular events for patients with chronic hyperglycemia begins with prediabetes. Not only because of additional risk factors, but because both increased insulin levels and peripheral insulin resistance trigger an important cascade of changes that alter the contractile, vascular, and electrical structure of the heart. Once the diagnosis of type 2 diabetes has been made, it is estimated that approximately 8 to 10 years of hyperglycemia have passed [2,3].

Diabetes encompasses a spectrum of cardiovascular complications, including coronary artery disease, limb ischemia, cerebrovascular disease, ischemic heart disease, and heart failure. Patients with diabetes fall into heart failure stage A, as classified by the American College of Cardiology [2]. A particularly contentious cardiovascular complication of diabetes is diabetic cardiomyopathy, characterized by cardiac dysfunction and heart failure in the absence of coronary artery disease, hypertension, and other valvular heart disease [4]. This condition is marked by cardiac remodeling, featuring foci of fibrosis and parenchymal fatty dystrophy [2].

## 3. Type 2 Diabetes Mellitus and the Endothelium

Insulin primarily affects energy storage and distribution in suitable tissues, but it also has important hemodynamic effects. At physiological levels, insulin promotes the release of nitric oxide and endothelin-1 facilitating the vasorelaxation and vasoconstriction states to guarantee the recruitment of vascular vessels in energy demand scenarios (e.g., skeletal muscle during aerobic exercise). Insulin resistance drives the loss of vasorelaxation and vessel recruitment through impairment of the PI3K/Akt and MAPK signaling pathways, which allows a rapid and aggressive progression of atherosclerosis and a suitable scene for severe vasoconstriction events [5].

Therefore, the role of endothelium in type 2 diabetes cardiovascular disease burden has been explored, and it has become a therapeutic target to prevent diabetes-related complications with a particular spotlight on how new antidiabetic drugs through their pleiotropic effects can restore insulin-dependent endothelium signaling.

## 4. Coronary Artery Disease

Chronic hyperglycemia is responsible for inducing the production of AGEs (Advanced Glycation End Products). The interaction between AGE and RAGEs (Advanced Glycation End Products Receptors) has severe consequences as it exerts pro-inflammatory effects, generates ROS (Reactive Oxygen Species), and expresses adhesion molecules, such as vascular cell adhesion molecule 1 (VCAM-1) and intercellular cell adhesion molecule 1 (ICAM-1), which promote the entry of monocytes into the subendothelium [6]. This, in turn, leads to a decrease in vasodilation by decreasing nitric oxide (NO), enhances vasoconstriction by increasing endothelin-1, and enhances macrophage phagocytosis by expressing the scavenger receptors, including a cluster of differentiation-36 (CD36) and SR class A1 [7,8]. 

The modification of low-density lipoprotein (LDL) is equally problematic. The LDL molecule is first desialylated to form small dense LDL. This is followed by oxidation or glycation of a small dense LDL, which favors the interaction with subendothelial proteoglycan, enhancing the retention time of LDL, the LDL phagocytosis by the macrophages to form the lipid-laden foam cells, and the release of proinflammatory cytokines, such as TNF-α, IL-1β, IL-6, and matrix metalloproteinases (MMPs), by foam cells. [8].

**Figure 1:**
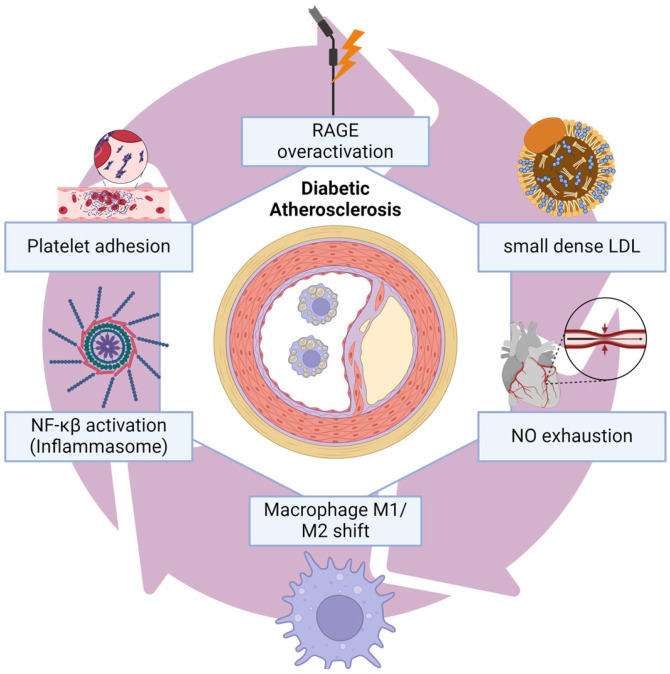
Diabetic atherosclerosis and coronary disease mechanisms. Chronic exposure to hyperglycemia leads to the overactivation of diverse adverse damage pathways all converging in inflammation that goes through the macrophage phenotype shifting, favoring low-grade local inflammation, NLPR3-induced inflammasome activation and accelerated fibrosis, depletion of NO and decreased vasorelaxation, a more aggressive deposition of small dense LDL in coronary endothelium, and a prothrombotic state due lipotoxicity and macrophage shifting and RAGE overaction that induces pro-apoptotic pathways and synthesis of pro-inflammatory signals. NO: Nitric Oxide; RAGE: Advanced Glycation End Products Receptors; LDL: Low-Density Lipoprotein.

Hyperglycaemia is known to cause changes in gene expression through epigenetic modifications, which do not involve changes in the DNA sequence. These modifications include the formation of AGEs, increased oxidative stress, upregulation of polyol pathway and hexosamine biosynthetic pathway, enhanced PKC (protein kinase C) and TGF-β-smad-MAPK signaling, and increased expression of NF-κβ dependent monocyte chemotactic protein-1 (MCP-1) and VCAM-1. These changes have been linked to the development of endothelial dysfunction and atherosclerosis [8,9]. Dysregulated expression of microRNAs (miRNAs) and lncRNAs are also involved in various stages of atherosclerosis development, including lipid metabolism, endothelial dysfunction, VSMC (vascular smooth muscle cells) phenotypic switch, macrophage phenotypic switch, platelet reactivity/aggregation, and cardiomyocyte differentiation and apoptosis. In particular, the macrophage phenotypic switch from anti-inflammatory M2 to proinflammatory M1 phenotype results in the release of various cytokines, chemokines, and ICAM-1, which are mediated by increased NF-κβ and activator protein-1 (AP-1) signaling. This switch can be triggered by AGEs, polyols, O-GlcNAc, PKC, lipotoxicity, oxidative stress, mitochondrial dysfunction, and epigenetic modifications [3,8,9]. Coronary disease mechanisms can be seen in Figure 1 [8,10].

Intestinal microbiota can also influence coronary heart disease. Phenylacetylglutamine (PAGln) is a metabolite formed by the gut microbiota by conjugating glutamine and phenylacetate. It has been linked to increased platelet activity and a higher risk of thrombosis [11]. Recent studies have found that PAGln serum levels are correlated with the severity of coronary atherosclerosis and the incidence of cardiovascular disease (CVD) and major adverse cardiac events (MACEs). Additionally, high PAGln levels are independently associated with an increased risk of coronary in-stent restenosis, a condition that worsens the prognosis for patients with coronary artery disease (CAD) [12].

Adiponectin has an interesting relationship with the severity of coronary heart disease, especially in type 2 diabetes. White adipocytes secrete this hormone, and it is classically known as an insulin-sensitizing hormone, but evidence has shown that it produces anti-inflammatory, anti-atherosclerotic, and cardioprotective effects. The adiponectin receptor 1 (AdipoR1) is widely expressed in cardiomyocytes and endothelial and vascular smooth muscle cells. In type 2 diabetes, the secretion of adiponectin by adipose tissue is decreased, and this is correlated with a higher risk of cardiovascular events in diabetic patients [13,14,15].

In the signaling pathways of atherosclerotic disease, adiponectin promotes the macrophages shifting to M2 phenotype (anti-inflammatory profile) and the proper differentiation of endothelial cells, while inhibiting the expression of atherogenic molecules (VCAM-1 and ICAM-1), and reducing LDL oxidation, platelet aggregation, and the proliferation and migration of vascular smooth muscle cells in the intima of coronary vessels. On the other hand, adiponectin also protects cardiomyocytes through the AMPK signaling pathway, preventing apoptosis by the inhibition TFG-β signaling and recovering the autophagy downstream effects, thus preventing cardiac remodeling and fibrosis [13].

## 5. Diabetic Cardiomyopathy

**Figure 2:**
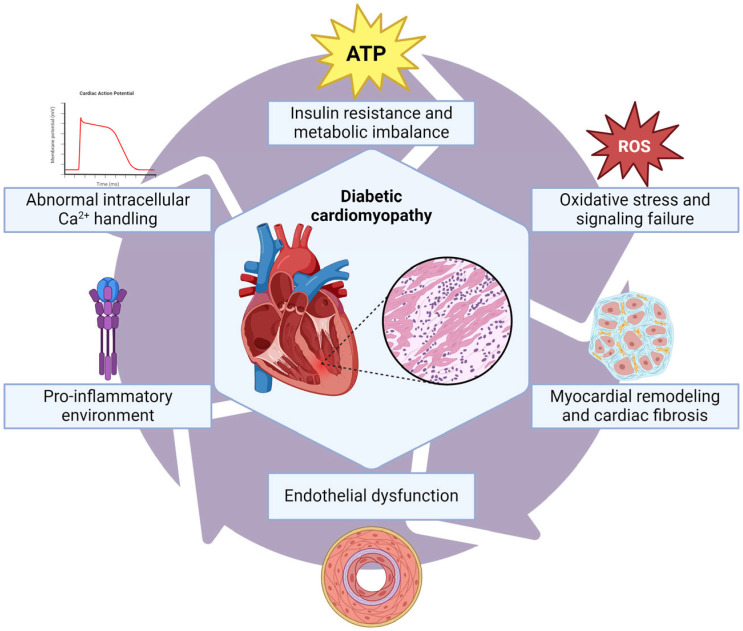
Type 2 Diabetes-related cardiomyopathy summary of pathophysiological pathways. The effect of chronic and persistent high glucose levels drives the cardiomyocyte to ROS and glucosamines production through the polyol and hexosamine pathways, which activates AGEs formation and NF-κβ inflammatory pathway, thereby inducing myocardial remodeling and cardiac fibrosis through TFG-β signaling; furthermore, the late and progressive insulin resistance in the cardiomyocyte leads to lipotoxicity by the increased uptake of fatty acids due decreased glucose uptake; moreover, the exceeded lipid oxidation process favors PKC dysfunction and autophagy, worsening cardiac fibrosis and producing an uncoupling of the excitation-contraction process with an abnormal intracellular calcium handling.

The pathophysiology of diabetic cardiomyopathy involves a multitude of factors, and despite extensive research, there is no singular pathognomonic molecular alteration that comprehensively explains this complex phenomenon [4]. Notably, several molecular markers have been identified (Figure 2).

*Insulin Resistance and Metabolic Imbalance*: Cardiac hypertrophy in diabetic cardiomyopathy is associated with insulin resistance and hyperinsulinemia [16]. Insulin receptor dysfunction promotes an asymmetric availability of fatty acid translocase (FAT) and GLUT4, with an excessive accumulation of fatty acids (FAs) in cardiac tissue, and produces saturation of mitochondrial capacity to oxidize FAs, which leads to increased availability of lipid intermediates as diacylglycerol (DAG), ceramides, and acylcarnitines [17]. These lipid intermediates induce the peroxidation of lysosome membranes and inhibit autolysosome formation, which is responsible for the degradation of abnormal intracellular proteins, damaged structures, and the recycling of synthesis substrates [18]. The failure of the autophagy pathway and the excessive ROS production led to worsening insulin resistance and inhibition of the mTOR pathway. Additionally, limited myocardial utilization of FAs (due to mitochondrial and autophagy functioning failure) in diabetes contributes to myocardial dysfunction, potentially explaining the improved outcomes in heart failure with diabetes seen with SGLT-2 inhibitors, which increase ketone bodies and provide an alternative energy source to the failing heart [9,19].

*Oxidative Stress*: Increased fatty acid uptake through FAT and decreased GLUT4-mediated glucose uptake due to insulin resistance restricts the cardiomyocytes that rely on the lipid oxidation pathway to maintain energy supply. The disruption of glucose metabolism enhances alternative metabolic routes as the polyol and hexosamine pathways result in the production of AGEs and O-GlcNAc, with a subsequent increase in ROS and endoplasmic reticulum (ER) stress [20]. Another ROS source is the activation of the NOX protein family via protein kinase C (PKC), due to increased mitochondrial lipid oxidation by DAG. The ROS and AGEs can further interact with RAGE and activate the GSK-3β, ERK 1/2, and p38 transduction pathways, promoting nuclear translocation of NF-κβ and inducing proinflammatory cytokine production as TNF-α and IL-6 and IL-8 [2,20].

*Myocardial Remodeling*: The energy expenditure imbalance, ER stress, and ROS excessive accumulation drive cardiomyocytes into apoptosis; the released DAMPs (damage-associated molecular patterns) It induces the activation of cardiac fibroblasts and the recruitment of immune cells, and these fibroblasts transdifferentiate into myofibroblasts. This phenomenon is potentiated via TGF-β released by endothelial cells through AGEs and ROS stimulation; hyperglycemia worsens this scenario through increased expression of TGF-β via Akt decreased activity and increased expression of FoxO1, which enhances expression of α-SMA (alpha-smooth muscle actin), and therefore, more dysfunctional collagen synthesis and extracellular matrix degradation and progression of cardiac fibrosis [21]. Furthermore, hyperglycemia triggers an aberrant activation of the renin-angiotensin-aldosterone system with increased activation of AT-1R (angiotensin-1 receptor) and MR (mineralocorticoid receptor) due to angiotensin II (AT II) and aldosterone both increasing expression of TGF-β/Smad signaling [22]. AT II inhibits the autophagy pathway to induce expression of myosin heavy chain gene (MHC) and shifts from α-MHC to β-MHC, thus promoting a transitional adaptative compensatory hypertrophy in the viable cardiomyocytes after cardiac injury, but with persistent activation due to hyperglycemia, this leads to cardiac fibrosis [23]. Meanwhile, aldosterone stimulates the LC3-II/LC3-I expression, promoting and subsequently producing a dysregulation of autophagosome formation and increasing the cardiac wall thickness, collagen synthesis, and diastolic dysfunction [24]. Overexpression of cardiomyocyte hypertrophy genes, including IGF1-R, BNP, and the myosin heavy chain gene, as well as cardiac overexpression of PKC, contributes to cardiac hypertrophy and fibrosis. Inflammatory mediators further induce cardiomyocyte hypertrophy [20].

*Endothelial Dysfunction*: Hyperglycemia-associated endothelial dysfunction results from an imbalance in oxidative pathways, reducing endothelial nitric oxide synthase (eNOS) and causing decreased nitric oxide (NO) production [25]. Endothelial cells’ main glucose uptake mechanism is GLUT1, and in hyperglycemia conditions, this drives ROS production and ER stress, which promotes calcium-impaired signaling and less efficient vasodilatation [26,27]. Endothelin-1 (ET-1) is a strong agonist of vasoconstriction in vascular smooth cells via PLC/IP3/Ca^2+^/PKC but in cardiac endothelial cells, induces nitric oxide production and prevents overstimulation of TNF-α induced apoptosis. PKC activation by DAG leads to increased vascular permeability and leukocyte migration, with further endovascular inflammation; the persistent proinflammatory signaling depletes NO and exerts ET-1-mediated vasoconstriction over NO secretion by cardiac endothelial cells. The expression and mRNA levels of eNOS are not compromised in hyperglycemia conditions but are inhibited by ROS and increased the availability of asymmetric dimethylarginine (ADMA); on the contrary, inducible nitric oxide synthase (iNOS) has an aberrant expression and activity, which leads to cardiomyocyte death due to caspase-3 nitrosylation and excitation-contraction uncoupling through myosin light chain (MLC) dysfunction and promotes cardiac wall stiffness, depletion of vasodilatation mechanisms, and diastolic dysfunction [21,25,27].

*Pro-Inflammatory Environment*: Low-grade inflammation is a hallmark of diabetes [10]. Locally, increased pro-inflammatory cytokines, TNF-α, IL-6, and IL-8, are mediated by the activation of the NF-κβ signaling pathway, triggered by ROS production, PKC overactivation, and RAGE activation. Activation of proinflammatory T helper cells and macrophage polarization to inflammatory profiles (M1) occurs in the insulin resistance scenario; these activated immune cells secrete cytokines aggravating the local inflammation [28]. Further, increased vascular permeability due to hyperglycemia leads to leukocyte infiltration of the cardiac interstitium, DAMPs signaling by mitochondrial stress via lipotoxicity, and cardiomyocyte apoptosis induce activation of TLR2 and TLR4 along with increased NLRP3, driving the pro-caspase-1 recruitment for inflammasome formation, amplifying myocardial cytokine production, macrophage infiltration, and cardiomyocyte apoptosis and peripheral cardiac fibrosis [26,29,30,31].

Gut microbiota plays a significant role in influencing heart disease through its metabolic and immunological functions. Its interaction with the host contributes to metabolic processes and can trigger systemic inflammation [32]. Trimethylamine N-Oxide (TMAO) is a harmful metabolite produced by the gut microbiota from food sources. In the liver, TMA is oxidized to TMAO by flavin-monooxygenase-3 (FMO3) and then released into the systemic circulation. It also triggers signaling pathways, such as the phosphorylation of extracellular signal-regulated protein kinases (ERK1/2) and Jun N-terminal kinase (JNK), leading to increased platelet activity, endothelial damage, and atherosclerotic plaque formation [33]. In vitro studies have shown that TMAO induces the expression of genes linked to inflammation, such as cyclooxygenase 2 and interleukin-6, and promotes leukocyte adhesion to the vascular endothelium, causing inflammatory damage to the blood vessels [34].

*Abnormal Intracellular Calcium Handling*: Diabetic cardiomyopathy involves decreased calcium uptake through a reduction in the membrane availability of L-type Ca^2+^ channels, increased hyperphosphorylation, and dysfunction by lipid peroxidation of Ryanodine receptors (RyR2), which leads to inefficient calcium homeostasis and resulting in arrhythmogenic waves mediated by the leak of Ca^2+^ from the sarcoplasmic reticulum (SR) [35]. Additionally, the SR Ca^2+^-ATPase (SERCA) is downregulated in insulin resistance and hyperglycemia conditions with inhibition of ATP-binding sites by ROS and O-GlcNAc, which induces post-translation modifications in calcium transporters and signaling proteins related to survival and metabolism pathways. Chronic hyperglycemia is associated with an increased ratio of the unphosphorylated phospholamban state (PLB), thus increasing inhibition of the SERCA, resulting in decreased Ca^2+^ uptake by the SR leading to diminished contractility and loss of lusitropic response in the cardiomyocytes, thereby decreasing the stroke volume [36]. Along with these findings, there is a prolonged refractory period phase, shortening of the systolic phases, and prolonging of diastolic phases, culminating in impaired relaxation. Calcium cytoplasmic overload induces cardiomyocyte apoptosis [36,37,38].

**Figure 3:**
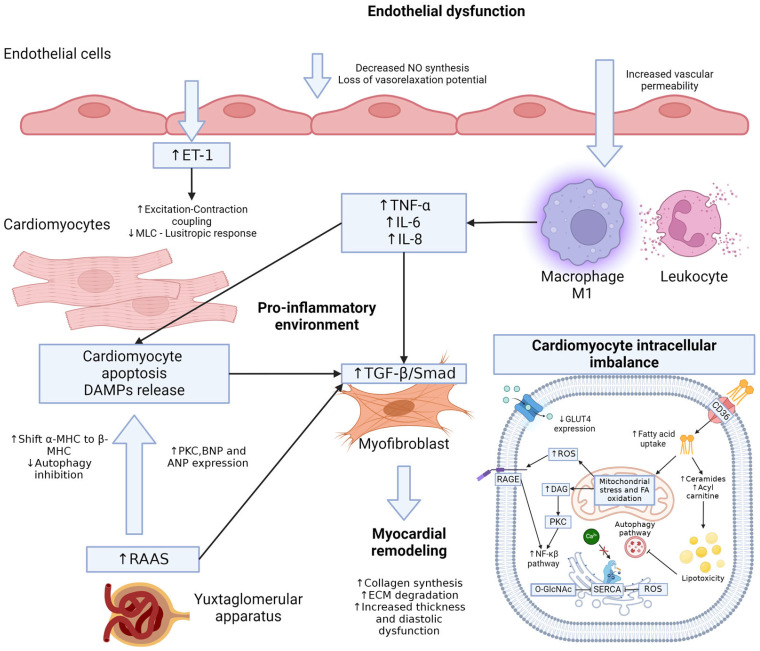
Molecular mechanisms of type 2 diabetes cardiomyopathy associated remodeling and fibrosis. The initial endothelial dysfunction due to chronic hyperglycemia and insulin resistance leads to NO depletion and loss of vasorelaxation capabilities. Additionally, the disruption of the endothelial cell adhesion process favors increased vascular permeability, which allows the migration of M1 macrophages and leukocytes that promote myocardial interstitial inflammation and increased ET-1 signaling. The M1 macrophages secrete pro-inflammatory signals that promote cardiomyocyte apoptosis and DAMPs release converging in an overactivated TGF-β/smad pathway, which leads to myocardial remodeling with ECM degradation and subsequent fibrosis, thereby producing diastolic dysfunction (HFpEF). The RASS affected due to chronic hyperglycemia increases the stimulation of cardiac remodeling pathways through BNP, ANP, and PKC expression in cardiomyocytes. The diabetic cardiomyocyte with decreased expression of GLUT4 satisfies its energy demand through fatty acids but with increased ceramides and acylcarnitine synthesis that produce lipotoxicity, which inhibits autophagy and perpetuates dysfunctional receptors and other critical proteins for cardiomyocyte contractility. Mitochondrial stress, due to fatty acid oxidation, induces ROS and DAG production with consequent RAGE and PKC activation with secondary inflammatory signals increased expression. All these cellular events converge in the dysfunction of calcium intracellular handling with deficient contractile function and the hallmarks of HFpEF. NO: Nitric Oxide; ET-1: Endothelin-1; MLC: Myosin Light Chain; DAMP: Damage Associated Molecular Patterns; MHC: Myosin Heavy Chain; PKC: Protein Kinase C; BNP: Brain Natriuretic Peptide; ANP: Atrial Natriuretic Peptide; TFG-β: Transforming Growth Factor Beta; TNF-α: Tumoral Necrosis Factor Alfa; DAG: Diacylglycerol; SERCA: Sarcoplasmic/Endoplasmic Reticulum Ca^2+^-ATPase; ROS: Reactive Oxygen Species; RAAS: Renin-Angiotensin-Aldosterone System.

Additionally, epigenetic changes, such as hypermethylation, demethylation, and histone modifications, play a role in diabetic cardiomyopathy [2,4]. Alterations in miRNAs, exemplified by miR-320, and changes in lncRNAs contribute to inflammation, myocardial injury, cardiac hypertrophy, and other diabetic vascular complications [39]. Targeting lncRNAs with future therapies involving RNA interference or CRISPR/Cas9 gene editing holds promise for addressing these intricate molecular mechanisms in diabetic cardiomyopathy [4,39] (Figure 3).

## 6. Heart Failure

The mechanisms by which heart failure (HF) develops in patients with type 2 diabetes are beyond glycemic control. On the one hand, the use of energy substrates in the heart changes with increased utilization of fatty acids and ketone bodies. This is associated with an increase in the hexosamine pathway flux, leading to more glucosamine production, which is ultimately used by O-GlcNAc transferase to generate posttranslational modifications on a diverse array of cellular proteins via a process known as O-GlcNAcylation. This process is correlated with mitochondrial dysfunction, ventricular hypertrophy, and heart failure. Mitochondrial function is not only affected by reactive oxygen species but also by lipotoxicity derivatives such as ceramides. The reuptake of calcium via SERCA-2 (sarcoplasmic/endoplasmic reticulum Ca^2+^-ATPase-2) into the sarcoplasmic reticulum (SR) is an energy-dependent process that is compromised in heart failure [40]. 

ER stress has been linked to myocardial inflammation and HF. Hyperglycemia-associated increases in ER stress correlate with oxidative stress. The genetic factor CHOP (C/EBP homologous protein) operates downstream of eIF2α phosphorylation, which is elevated by ER stress and is associated with pressure overload-induced cardiac hypertrophy. Toll-like receptors (TLRs) are expressed in cardiomyocytes and are critical mediators of immune signaling. Elevated levels of glucose and free fatty acids (FFAs) activate TLR2 and TLR4, contributing to pro-inflammatory responses. In patients with type 2 diabetes, hyperglycemia upregulates TLR2 and TLR4 expression in monocytes. Pharmacologic antagonism of TLRs mitigates NF-κB activation, reduces leukocyte infiltration, and improves myocardial contractile function in murine models subjected to ischemia/reperfusion injury [41]. 

A well-characterized downstream mediator in insulin signaling pathways relevant to HF pathogenesis involves the Forkhead box O (FOXO) family of transcription factors. FOXO proteins are implicated in the pathophysiology of HF associated with obesity and diabetes mellitus. Phosphorylation of FOXO1 (at Ser265) and FOXO3 (at Thr32 and Ser235) by Akt facilitates their translocation from the nucleus to the cytoplasm, where they are sequestered by binding to 14-3-3 proteins, effectively attenuating their transcriptional activity. In contrast, FOXO activation is induced by the disruption of the 14-3-3 interaction via acetylation mediated by p300 or AMPK, or through SIRT1-mediated deacetylation of FOXO1, leading to nuclear translocation and enhanced transcriptional activity. Nuclear-localized FOXO1 and FOXO3 promote cardiac atrophy and autophagy, whereas inhibition of FOXO3 is associated with pathological hypertrophy [42,43,44].

Constitutive activation of PI3K, although it promotes compensated hypertrophy, also induces desensitization of insulin-mediated glucose uptake. Notably, transient Akt activation has been shown to produce reversible hypertrophy; however, sustained Akt activation leads to progressive heart failure and delayed coronary angiogenesis. When Akt signaling was subsequently downregulated in these failing myocardium models, an accelerated progression to mortality was observed [44,45]. In vascular cells, ROS induces sustained epigenetic activation of the NF-κB p65 subunit. Histone 3 lysine 4 (H3K4) methylation has been identified as a critical epigenetic modification that upregulates p65 transcription, thereby enhancing the expression of downstream pro-inflammatory genes [46]. 

In the development of HF with preserved ejection fraction (HFpEF), although reactive oxygen species have a short half-life, they produce 4-hydroxy-2-nonenal (4HNE), like other reactive carbonyls, through lipid peroxidation. High levels of 4HNE have been documented in cardiac tissue in the presence of type 2 diabetes. In myocardial cells, 4HNE is detoxified by glutathione, aldehyde dehydrogenases (ALDHs), and aldose reductase. Elevated levels of 4HNE are associated with reduced ALDH2 activity, and animal models have shown that the E487K genetic mutation in ALDH2 can exacerbate characteristics of heart failure with preserved ejection fraction [47]. Detoxification of 4-HNE can also occur through a glutathione-mediated pathway. In myocardial cells from isolated perfused rat hearts, 4-HNE reacts with reduced glutathione (GSH) to form the glutathione S-conjugate of 4-hydroxynonenal (GS-HNE). To date, a potential link has been suggested between 4-HNE and pathological conditions, such as atherosclerosis, hypertrophy, cardiomyopathy, myocardial ischemia-reperfusion injury, and arrhythmias [48]. 

A critical signaling role of ALDH2 involves the peroxisome proliferator-activated receptor-γ coactivator (PGC-1α). Insulin resistance associated with diabetes promotes increased acetylation of PGC-1α, a coactivator essential for oxidative phosphorylation, mitochondrial biogenesis, and cardiac development. When acetylated PGC-1α accumulates at high levels, intracellular Ca^2+^ regulation is impaired, ROS levels are significantly elevated, and cardiac contractility is compromised. In response, mitochondrial ALDH2 facilitates the deacetylation of PGC-1α via Sirt3, a deacetylase that PGC-1α employs as a critical mediator of mitochondrial metabolic regulation. Mitochondrial dysfunction plays a central role in all processes, leading to heart failure [49]. 

## 7. Arrhythmias

TIC (tachycardia-induced cardiopathy) is defined as a ventricular dysfunction resulting from a prolonged elevated heart rate or irregular rhythm, reversible upon control of the arrhythmia [50,51]. TIC is usually seen in patients with no previous structural heart disease, although it may be responsible for ventricular dysfunction aggravation in those with underlying heart disease. High or irregular ventricular beats result in atrial dilatation, ventricular dilatation, or both, plus mitral regurgitation [52]. TIC is frequently misdiagnosed as dilated idiopathic cardiomyopathy, in part due to the major reported symptoms being related to heart failure (47% of patients) rather than palpitations (29%) [53], making TIC diagnoses underdiagnosed, and therefore, underestimated [52].

Most information regarding TIC is driven by atrial fibrillation (AF), atrial flutter, and atrial tachycardia [52]. Up to 75% of patients with AF and heart failure recover left ventricular function (and improve cardiovascular outcomes) after pulmonary veins isolation as rhythm control or atrioventricular nodal ablation for rate control [54,55,56]. Another strong association is seen in permanent junctional tachycardia (20–50% of patients develop TIC) followed by incessant atrial tachycardia (prevalence of TIC of 37%). Less frequently it has been reported as related to premature ventricular complexes, ventricular pacing, and other forms of supraventricular and ventricular tachycardia [56].

Tachycardia or irregular heartbeat is associated with elevated ventricular filling pressures, decreased contractility, right and left ventricular wall thinning, and heart failure with neurohormonal activation [51,52]. Abnormal calcium handling reduced cellular energy storing, and abnormal energy use have been proposed as the underlying mechanisms responsible for this syndrome [57]. Cellular changes include loss of myocytes, cellular elongation, myofibril misalignment, and loss of sarcomere register, which may be due to derangement of the extracellular matrix. Upon normalization of heart rate, TIC can be partially or fully reversed in weeks or months, typically marked by a considerable improvement in ejection fraction. However, subtle and persistent damages after recovery have also been reported in patients with TIC [57,58,59].

The relationship between TIC and diabetes mellitus (DM) is hard to clarify due to underdiagnosis of TIC, poor referral to effective rhythm control in patients with heart failure, and heterogenicity of disease in diabetes (e.g., severity, glycemic control, years of disease, comorbidities, obesity, or exposure to cardiovascular modifying drugs). However, some psychopathological assumptions might be made, especially in the case of AF and DM is a known risk factor for AF [60].

Cardiac autonomic neuropathy (CAN) is associated with the impairment of autonomic control of the cardiovascular system and is a major cause of silent cardiovascular events. The prevalence of CAN is highly variable and underdiagnosed and is estimated to be between 20 and 65% [61]. The vagus nerve is involved early, resulting in parasympathetic denervation and sympathetic predominance. Sympathetic denervation results in unresponsiveness of heart rate and blood pressure to stress [61,62].

Hyperglycemia and dyslipidemia are the main drivers of CAN. In the presence of hyperglycemia, glucose enters the Schwann cells through glucose transporter 3 (GLUT3). Excess glucose undergoes glycolysis, and pyruvate exceeds the capacity of the tricarboxylic acid (TCA) cycle, resulting in a shift to anaerobic metabolism and accumulation of lactate. Lactate is shuttled from Schwann cells into axons, resulting in mitochondrial dysfunction and axonal degeneration [8].

Hyperglycemia results in excessive activation of the electron transport chain, leading to mitochondrial dysfunction, ROS generation, oxidative stress, DNA damage, and activation of polyadenosine diphosphate ribose polymerase. The last, in turn, inhibits glyceraldehyde-3-phosphate dehydrogenase resulting in the accumulation of glycolytic metabolites, with upregulation of polyol, hexosamine, and DAG and PKC pathways, as well as generation of AGEs [63,64]. The AGE-RAGE interactions, oxidative stress, endoplasmic reticulum (ER) stress, and upregulated non-glycolytic pathways result in endothelial dysfunction characterized by impaired vasodilation mediated by decreased NO bioavailability, increased ET-1, increased PAI-1, and aberrant angiogenesis [8].

Sinus chronic tachycardia is not the only rhythm disturbance. The risk of AF development in patients with DM has been established by large studies and metanalyses showing a clear link between AF and DM. Based on the association between the two medical conditions and the high risk of cardiovascular morbidity and mortality that their combination presents, the literature has concluded that underlying pathophysiology is related to structural, electrical–mechanical, and autonomic remodeling, as well as metabolic parameters [60].

**Figure 4:**
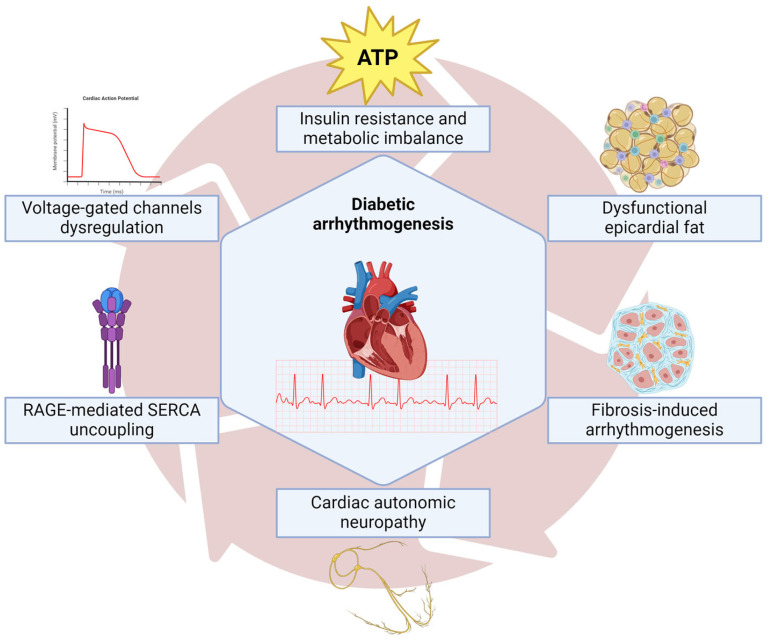
Summary of pathophysiological mechanisms related to diabetic arrhythmogenesis. The origin of the spectrum of diabetes-heart related is insulin resistance and metabolic imbalance that induces excitability and conduction dysfunction due to impairment in energy supply and ion flux; additionally, the epicardial fat in diabetic patients releases fatty acids that negatively regulate voltage-gated channels facilitating arrhythmogenic foci; furthermore, the cardiac autonomic neuropathy, represented mainly by the vagus nerve loss of stimuli on the cardiomyocytes, worsens the chronotropic and dromotropic control, increasing the probability of tachycardia-induced cardiomyopathy and atrial fibrillation. Progressive cardiac fibrosis is another mechanism that induces re-entry phenomenon and loss of atrial-ventricular communication with increased risk of ventricular arrhythmias and finally, the RAGE signaling pathway that enhances uncoupling of SERCA activity with a dysfunctional intracellular calcium handling, thus producing an ineffective excitation-contraction process.

Chronic hyperglycemia creates a substrate for atrial remodeling and initiating AF, the most prominent atrial dilatation and fibrosis. Hyperglycemia is also associated with enhanced angiotensin II signaling and reactive oxygen species production, linked to inflammation and pro-fibrotic pathways [65,66,67]. Furthermore, high glucose levels can enhance fibrosis through the production of advanced glycation end-products, which can regulate cardiac fibroblasts by activating their surface receptors [68]. Last, adipokines, such as leptin and frizzled-related protein 5 produced in the epicardial fat layer, have been implicated in the pathophysiology of AF in diabetic patients [69]. Adipokines may represent important biomarkers in the risk prediction and management of diabetic complications since they are implicated in mitochondrial energetics, oxidative stress, and apoptosis pathways [70]. In Figure 4 we present a summary of diabetic arrythmogenesis.

## 8. Therapeutic Insights

The principle for the development of new antidiabetic drugs is cardiovascular safety; furthermore, these new therapeutic options must pursue the prevention of cardiovascular events and other end-organ damage complications related to the disease, beyond the HbA1C-centered approach [71]. Several studies are strengthening the need for an early intensive intervention in glycemic control but with interventions without increasing the risk of hypoglycemia or cardiovascular events [72].

Metformin stands as the first-line therapy for diabetes due to its cardiovascular safety, cost-effectiveness, and glycemic control effects. In a murine model, there is evidence of improving left ventricular function regardless of glycemic control; nevertheless, in a phase IV clinical trial, there was no systolic improvement [73]. This mechanism appears to be explained due to the AMP-activated protein kinase (AMPK) activation, thus improving energy efficiency in the cardiomyocyte through recovering autophagy pathways counteracting insulin resistance effects [74]. Evidence could sufficiently explain whether metformin can be seen as a cardiovascular protective intervention but is not sufficient to attend to the therapeutic goals of diabetic patients with or without established cardiovascular disease.

Sodium-glucose cotransporter 2 inhibitors (iSGLT2s) are known to prevent heart failure-related hospitalizations and death due to heart failure. The understanding of the mechanisms of iSGLT2 benefits is still in process, but recent findings about its cardiovascular effects showed that empagliflozin and dapagliflozin protect against lipotoxicity in human myeloid angiogenic cells and platelets [75]; additionally, iSGLT2 decreases sympathetic nerve activity and attenuates late sodium currents in cardiomyocytes and the metabolic precursor’s availability in the suprarenal medulla [76]. These results could explain how iSGLT2s produce their cardiovascular protective effect and reduce the risk of TIC in diabetic patients [77].

Glucagon-like peptide-1 (GLP-1) analogs have been an important inclusion to the diabetes treatment portfolio with rapid marked access through diverse regions of the world in a similar way to iSGLT2 and metformin. The main effects of these molecules are the recovery of β-cell function and a decrease in the probability of cell death in the natural history of diabetic disease; additionally, they enhance insulin sensitivity in peripheral tissues, lower LDL cholesterol levels, and reduce blood pressure, thereby contributing to a global attenuation of cardiovascular risk [78]. GLP-1 analogs can activate the Epac-2 (exchange protein activated by cyclic-AMP), and its function is to increase ANP secretion, therefore improving cardiomyocyte contractility [79]. Semaglutide has evidence of reducing TNF-α and proinflammatory biomarkers in vitro and in vivo studies, inhibiting myocardial fibrosis signaling pathways; this GLP-1 analog has evidence of preventing cardiovascular events in diabetic patients, and its effects on fat body mass could further improve other clinical challenges in the type 2 diabetes cardiovascular patients [80]. The pleiotropic effects of semaglutide on inflammation could explain how it can produce secondary prevention in MACE-related events, performing a synergy with its insulin sensitization and restoring the cardiomyocyte and endothelium insulin-dependent signaling.

## 9. Discussion

In recent decades, we have witnessed the evolution in the management of diabetes with therapies that reduce the complications of the disease, while at the same time advancing the understanding of the mechanisms that cause damage to the cardiomyocyte [1]. On the one hand, there has been significant progress in improving the understanding at the cellular level of mitochondrial dysfunction and how advanced glycation end products trigger an inflammatory cascade that causes damage at the cellular level [8]. Endothelial dysfunction is particularly important due to the loss of vasorelaxation and increased permeability, which, in the context of a pro-inflammatory environment combined with the neurohormonal response, leads to the apoptosis of cardiomyocytes [25]. This fibrosis is directly related to heart failure, vascular remodeling, and the disruption of the cardiac electrical conduction system [66]. On the other hand, the response induced by insulin to hyperglycemia generates an abnormal metabolic process in lipids, leading to small, dense, and oxidized low-density cholesterol. This cholesterol is not only more prone to precipitate and be retained in the vascular subendothelium but also triggers a greater inflammatory response, promoting a more complex atherosclerosis described in patients with type 2 diabetes [8]. The most underdiagnosed mechanism is related to the loss of control of cardiac automatism due to tachycardia, with reduced variability resulting from dysautonomia, and the higher prevalence of supraventricular arrhythmias. Greater awareness of this mechanism could strengthen the exploration in further studies [58]. 

This better understanding of the disease has led to a noticeable change in guidelines. Along with the greater availability of diagnostic techniques, such as ultrasonography, arterial Doppler, or coronary calcium score, and biomarkers, like pro-BNP, shifting away from the paradigm of not seeking cardiovascular complications in asymptomatic patients. Improved risk stratification and early detection in subclinical stages can change the disease trajectory [1].

Recently, the American Heart Association recommended using the term cardiorenal metabolic syndrome to describe the process related to the cardiovascular complications of the disease. Its stages are centered on the mechanisms that generate these complications, highlighting in stage 1, adipose tissue dysfunction, and in stage 2, the consequences of the inflammatory process in terms of hypertension, chronic hyperglycemia, alterations in the lipid profile, kidney damage, and metabolic consequences. Finally, stage 3 includes subclinical disease, and stage 4 establishes cardiovascular disease [81].

The development of new antidiabetic drugs focuses on cardiovascular safety and preventing cardiovascular events and complications beyond controlling HbA1C levels. Metformin’s benefits are linked to AMP-activated protein kinase (AMPK) activation, which enhances cardiomyocyte energy efficiency and counteracts insulin resistance by restoring autophagy pathways [73,74]. iSGLT2s are still being studied, and empagliflozin and dapagliflozin have been shown to protect against lipotoxicity in human myeloid angiogenic cells and platelets. Additionally, iSGLT2 inhibitors decrease sympathetic nerve activity, attenuate late sodium currents in cardiomyocytes, and reduce metabolic precursor availability in the adrenal medulla [75,76]. Glucagon-like peptide-1 (GLP-1) analogs help restore β-cell function, promote insulin sensitivity, reduce LDL cholesterol, and lower blood pressure, which together reduce overall cardiovascular risk. GLP-1 analogs activate the Epac-2 protein, increasing ANP secretion and improving cardiomyocyte contractility. Semaglutide, a GLP-1 analog, has been shown to reduce TNF-α and other proinflammatory biomarkers and inhibit myocardial fibrosis signaling pathways [78,79,80].

Although not all mechanisms have been fully elucidated, understanding the pathways through which the disease significantly decreases life expectancy and quality of life can contribute to developing therapies or early aggressive therapeutic interventions that could further reduce these adverse numbers and increase morbidity and mortality.
